# Mean Field Approximation for Biased Diffusion on Japanese Inter-Firm Trading Network

**DOI:** 10.1371/journal.pone.0091704

**Published:** 2014-03-13

**Authors:** Hayafumi Watanabe

**Affiliations:** Hottolink Inc., Chiyoda-ku, Tokyo, Japan; University of Warwick, United Kingdom

## Abstract

By analysing the financial data of firms across Japan, a nonlinear power law with an exponent of 1.3 was observed between the number of business partners (i.e. the degree of the inter-firm trading network) and sales. In a previous study using numerical simulations, we found that this scaling can be explained by both the money-transport model, where a firm (i.e. customer) distributes money to its out-edges (suppliers) in proportion to the in-degree of destinations, and by the correlations among the Japanese inter-firm trading network. However, in this previous study, we could not specifically identify what types of structure properties (or correlations) of the network determine the 1.3 exponent. In the present study, we more clearly elucidate the relationship between this nonlinear scaling and the network structure by applying mean-field approximation of the diffusion in a complex network to this money-transport model. Using theoretical analysis, we obtained the mean-field solution of the model and found that, in the case of the Japanese firms, the scaling exponent of 1.3 can be determined from the power law of the average degree of the nearest neighbours of the network with an exponent of −0.7.

## Introduction

Complex networks have been extensively studied over the last decade [1]–[3]. Problems of transport on complex networks, which are some of the most fundamental problems concerning the physics of complex networks, have also been intensively studied. For example, random walks on complex networks have been investigated from various viewpoints [4]–[6]. One application of transport on complex networks is PageRank, which corresponds to the steady-state density of transport caused by random walks on the Internet. PageRank is one of the most successful indices evaluating the importance of web pages and has been utilized by internet search engines.

Most studies regarding transport on complex networks have been based on theoretical approaches; however, recently the problems of actual transport on complex networks have also been studied by using massive data analysis. Examples of such problems include, airport traffic on the worldwide airport network [7], the number of trains on the Indian railway network [8], the flow of viewers on portal sites [9] and control flows on stock-ownership networks [10]–[12].

In a previous study, we analysed the empirical data from an inter-firm trading network, which consisted of approximately one million Japanese firms, and the sales of these firms (a sale corresponds to the total in-flow into a node) to investigate the actual transport phenomenon in a complex network [13]. This inter-firm trading network is known to be a typical complex network with a power law degree distribution [13], [14], a negative degree-degree correlation [13], [15], a small world property [14], community structures [16], power laws of money flows [15], [17] and an asymmetric behaviour of authorities and hubs explained by a network-evolution model based on the preferential attachment rule [18], [19]. To be more precise, we obtained the following results from Ref. [13]: (i) we found a non-trivial empirical power law with an exponent of 1.3 between the number of business partners in a firm and its sales by analysing the data; (ii) we introduced a simple money-transport model in which a firm (i.e. a customer) distributes money to its out-edges (i.e. suppliers) in proportion to the in-degree of the destinations; (iii) using numerical simulations, we found that the steady flow of the abovementioned inter-firm trading network derived from this model can approximately reproduce a power-law scaling with an exponent of 1.3 between the number of business partners (i.e. degrees of the network) and sales. Furthermore, the sales distribution of the actual firms obeys the power law distribution with an exponent of approximately 1. Moreover, the sales of individual firms derived from the money-transport model are shown to be proportional to the real sales on the average. However, we also showed that the simple random walk model (i.e., PageRank) in which a firm is assumed to evenly distribute to all its outgoing neighbours does not reproduce the empirical scaling with an exponent of 1.3. This result implies that PageRank does not correspond to the sales. Note that scaling with an exponent of 1.3 was observed are observed by analysing the abovementioned Japanese financial data not only between the sales and degrees but also between other important firm-size indicators, for instance, between sales and number of employees, between profits and number of employees, between profits and degrees, etc. [20].

In the previous study [13], we could not specifically identify what types of structure properties (or correlations) of the Japanese inter-firm trading network determine the 1.3 exponent, although we found that we need a particular network structure to be reproduced in our framework using numerical simulations. Therefore, the current study is devoted to the theoretical study of the results of Ref. [13]. In particular, we apply a mean-field approximation to the models to clarify how the exponents of the power-law relationships, specifically between the degree of the inter-firm trading network (i.e. the number of business partners) and sales depend on the network structure.

In this paper, we start in Section 2 by reviewing the models and the numerical results of the previous study. Next, we introduce the mean-field approximation for these models and reveal the relationships between the power-law exponent, models, and networks in Section 3. Finally, we summarize and present our conclusions in Section 4.

## Power Laws for Diffusion on Inter-Firm Trading Network

In this section, we review the results of Ref. [13]. To understand the empirical scaling with exponent 1.3 between degrees and sales, we introduced the following money-transport models [13].

Model-1 (Equi-partition model):
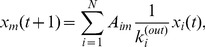
(1)


Model-2 (Weighted partition model):
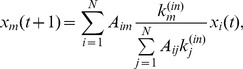
(2)where 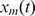 denotes the sales of node 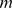 at time step 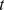, 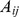 is an adjacency matrix, 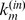 is the in-degree of node 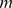, 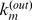 is the out-degree of node 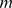 and 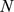 is the number of nodes on the network. Note that for 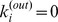 in Eq. (1) or 
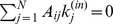
 in Eq. (2), we omit the contributions of the node 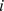.

In Model-1, a node representing a business firm is assumed to evenly distribute its total in-flow (i.e. sales) at time t to all its outgoing neighbours in the next time step. This transport is equivalent to the simple probability diffusion of the PageRank model in the case of no random spontaneous jumping [4]. However, for Model-2, a node distributes its sales to its outgoing neighbours in proportion to the destinations’ in-degrees. This model is one of the so-called biased random walk models [21].

We used two types of networks for numerical experiments to clarify the dependencies on the firm network [13]. The data set used in the generation of networks and the data analysis in Refs. [13], [20] was provided by Tokyo Shoko Research, Ltd. and contains approximately one million firms, which practically covers all active firms in Japan. For each firm, the data set contains the annual sales and a list of business partners in 2005, categorized into suppliers and customers [13], [14], [20].

The first network is the real firm network whose nodes are firms and whose edges are defined by the following rule: if firm 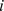 purchases goods and/or services from firm 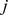, or, equivalently, if money flows from firm 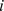 to firm 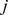, we connect node 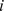 to node 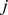 with a directed link (there are 961,318 nodes and 3,783,711 edges) [13], [14]. This network was generated by using the business partners data in the abovementioned data set. The firm network is a typical complex network whose main properties are as follows: (i)a power-law degree distribution with exponent 1.3, (ii)a negative degree-degree correlation and (iii)a small-world property (the mean distance between nodes is 5.62 and the maximum distance is 21) [13], [14], [16].

The second network is the shuffled firm network, which is an almost-uncorrelated network with the same degree distribution as the firm network generated by the Maslov-Sneppen algorithm [22], [23]. Note that we simulated the time evolution of the models on the largest strongly connected component (LSCC) for each network to neglect boundary effects. Then, total money is conserved, 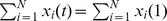 on the LSCCs of both networks.

For all combinations of models and networks, we obtained by numerical simulations the following power law for large 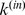 (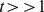) [13]:
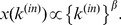
(3)


Here, 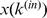 is the conditional mean of 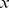 for a given 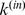 as a function of 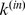 and is defined by
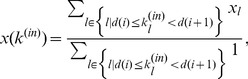
(4)where 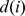 is the separator of box 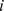, taken evenly in logarithmic space, e.g. 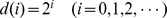, and 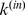 on the right-hand side is represented by the geometric mean 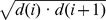. The exponent 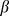 depends on both the network and model. These dependencies are summarized in Table 1. Note that Model 2 for the firm network reproduces the empirical scaling with exponent 1.3.

**Table 1:** Summary of exponents.

Model	Network	Exponent *β*
Equi-partition model	Firm network	1.0
	Shuffled network	1.0
Weighted partition model	Firm network	1.3
	Shuffled network	2.0
Sales (empirical data)	Firm network	1.3

## Mean-Field Approximation for Models

In this section, we apply the mean-field approximation to the models to understand the relationship between the exponents, models, and network structures as shown in Table 1. Here we neglect the directions of the networks for simplicity (scalings nearly identical to results presented in the previous section can be obtained by numerical simulation, regardless of whether or not the edge directions are neglected). By assuming 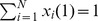, we can regard the models as Markov processes with an existing probability of 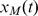,
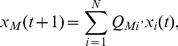
(5)where 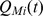 is the transition probability from node 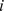 to node 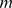 [13]. Note that although we neglect the direction of edges in this paper, the following discussion can be extended to the case of a directed network by adding some assumptions and calculations.

In general, one of the mean-field solutions of the Markov process defined by the transition matrix
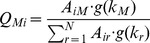
(6)is given in Ref. [24] by
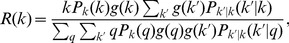
(7)where 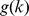 is the weight of the transition probability to a node with the degree 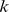, which is defined as a function of the degree 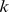, 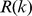 is the probability that the walker is at any node of degree 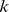 in the steady state, 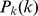 is a probability density function of degree 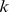 and 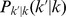 is the conditional probability that a node of degree 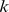 is connected to a node of degree 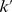. To obtain this solution, the authors of Ref. [24] employed mainly two approximations. First, they used the annealed network approximation where they regarded the sets of firms with common degrees in the original network as nodes of the approximated network, and the weighted edges of the approximated network are associated with a probability that two nodes of degrees 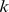 and 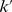 are connected,
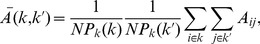
(8)where 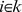 is denoted as a sum over the set of nodes of degree 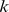 [24]. Second, they replaced 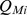 with average probability of an interaction between nodes of degree 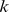 and 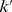, defined by



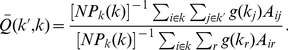
(9)Then, we can replaced the Markov process given by Eq. 5 with
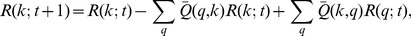
(10)where 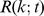 is the probability that the walker is at any node of degree 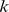 at time 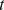. By substituting Eq. 7 into this equation and by using the degree detailed balance condition, 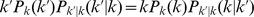, we confirm that Eq. 7 is a steady state of Eq. 10. More details on these approximations are available in Ref. [24]

Next, we apply Eq. 7 to our models given by Eqs. 1 and 2. In Model-1, the transition probability is uniform; that is, 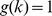 in Eq. 6. Therefore, the conditional mean of sales 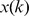 for the given degree 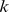 (i.e. 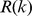 per node), is written as
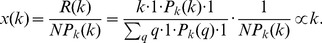
(11)


This scaling agrees with simulation results for Model-1 for the firm network and the shuffled network.

Similarly, by applying Eq. 7 to the case that 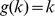 in Eq. 6 (corresponding to Model-2), we obtain
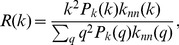
(12)where we denote the average degree of nearest neighbours as 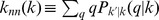 [25]. Thus, we find



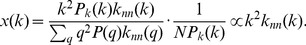
(13)From Fig. 1(a) in Ref. [13], we see that 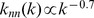 for a large degree 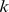 in the case of the firm network. We substitute this empirical result into Eq. 13 to obtain
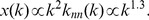
(14)


**Figure 1:**
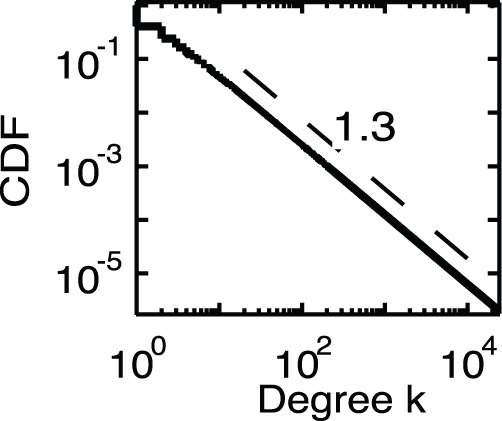
The cumulative distribution function of degree of the artificial networks. We confirm that degree obeys the power law distribution with exponent −1.3, namely, 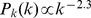.

This scaling corresponds to the simulation results for Model-2 for the firm network and empirical observation. Note that Eq. 13 implies that the exponents 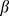 depend on the average degree of nearest neighbourhoods, 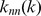 for Model-2.

For the shuffled network for Model-2, which is the uncorrelated network shown in Fig. 1(a) of Ref. [13] (i.e. 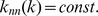), we obtain the following from Eq. 13:
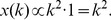
(15)


This equation agrees with the numerical result.

Finally, we numerically check the approximation by using different artificial networks that have power-law degree distributions with exponent 1.3 and power-law average degrees of nearest neighbours like the firm network. The artificial networks satisfy the following conditions:

- The degree obeys the power law distribution, 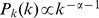.
- The average degree of the nearest neighbours is expressed as a power function of the degree, 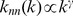.

To generate networks that satisfy the above conditions, we modify the configuration model as follows:

1. Assign the degree sequence, 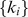, obeying the power law with exponent 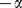 to nodes 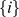. For example, 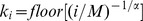 (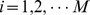), where 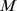 is the number of nodes and 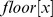 is the largest integer not greater than 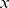.
2. Randomly sample a node denoted by the 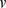 from the set of nodes that have the largest 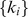.
3. Update 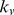; 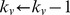.
4. Randomly sample a node denoted by 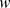 from all nodes except for node 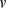 with probability proportional to 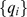, where 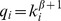.
5. Update 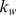; 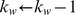.
6. Connect node 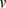 and 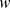 with an edge (undirected).
7. Repeat steps 2 through 6 until 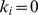 (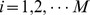).

**Figure 2:**
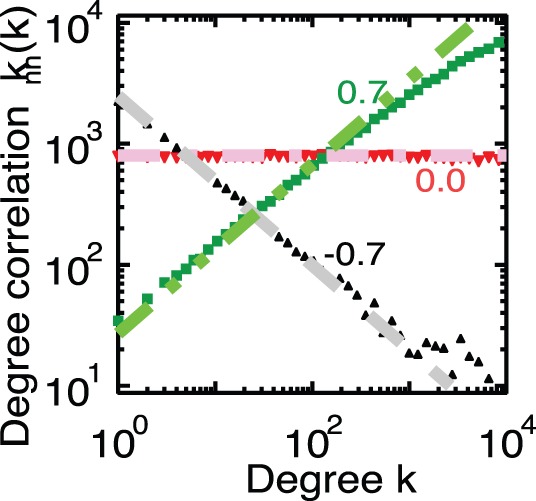
The averages degree of nearest neighbours as a function of degree of the artificial networks, 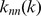. The black triangles indicate the network for 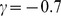, the red nablas for 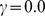 and the green squares for 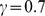. The black dashed line is proportional to 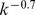, the red dash-dotted line is proportional to 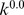 and the green dash-double-dotted line is proportional to 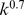.

**Figure 3:**
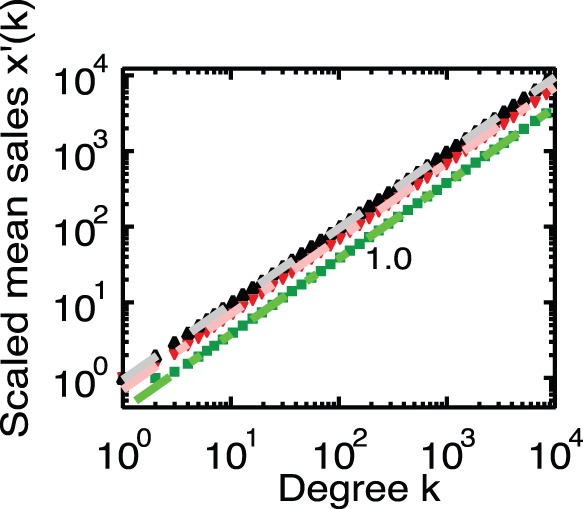
The mean of sales as a function of degree, 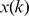, for Model-1. We plot the scaled values [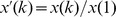]. The black triangles indicate the network for 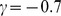, the red nablas for 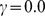 and the green squares for 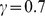. The mean of sales as a function of degree, 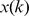, for Model-1. From this figure, we can confirm that 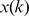 is proportional to 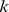 regardless of 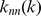. These results agree with the approximation given by Eq. 11, namely, 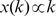.

**Figure 4:**
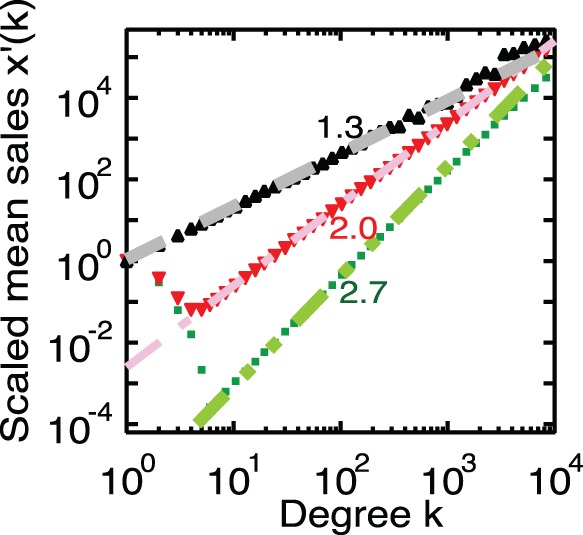
The mean of sales as a function of degree, 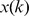, for Model-2. We plot the scaled values [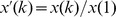]. The black triangles indicate the network for 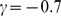, the red nablas for 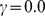, the green squares for 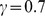, the black dashed line is proportional to 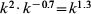, the red dash-dotted line is proportional to 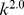 and the green dash-double-dotted line is proportional to 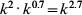. These results agree with the approximation given by Eq. 13; namely, 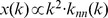. The result for 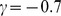 [i.e. 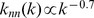] corresponds to empirical scaling with exponent 1.3.

Figures 1 and 2 show the cumulative distribution the function of degree, which corresponds to 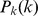 and the average degree of nearest neighbours as a function of degree 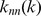 for the artificial network with parameter 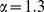 (empirical parameter), 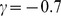 (empirical parameter), 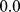 and 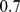. In addition, Figures 3 and 4 show the mean of the sales as a function of degree 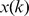, which is numerically derived from Eqs. 1 and 2 for the corresponding artificial networks. From these figures, we can confirm that all scaling exponents obtained numerically accord with the results of the approximations given by Eq. 11 for Model-1 and by Eq. 13 for Model-2. Moreover, from Figure 4, we confirm that only the case 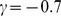, which is the empirical observation of 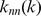 for the firm network, reproduces the empirical scaling 1.3.

## Conclusion and Discussion

In this study, by applying the mean-field approximation to the money-transport models given by Eqs. 1 and 2, we were able to consistently understand the relationships, between the power-law exponents, models and network structures summarised in Table 1. In particular, we presented the connections of the non-trivial power law scaling with an exponent of 1.3 (in Model-2 for the firm network) with the average degree of the nearest neighbours 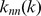, which is one of the cardinal features of a network. This result is one of the explanations for empirical scaling relationships with exponent 1.3 between the number of business partners and sales. Moreover, non-trivial empirical scaling with exponent 1.3 between sales 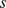 and number of employees 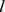, 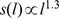, which is observed in Ref. [20], might be connected to the network structure. Because, roughly speaking, combining the scaling between sales 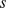 and degrees 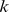, 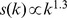, described in this study, and the trivial empirical scaling between number of employees 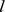 and degrees 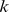, 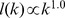 reported in Ref. [20], we obtain the scaling between employees and sales, 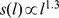.
